# Associations between klotho and telomere biology in high stress caregivers

**DOI:** 10.18632/aging.204961

**Published:** 2023-08-14

**Authors:** Ryan L. Brown, Elissa E. Epel, Jue Lin, Dena B. Dubal, Aric A. Prather

**Affiliations:** 1 Department of Psychiatry and Behavioral Sciences, University of California, San Francisco, CA 94107, USA; 2 Department of Biochemistry and Biophysics, University of California, San Francisco, CA 94107, USA; 3 Department of Neurology and Weill Institute of Neurosciences, University of California, San Francisco, CA 94107, USA

**Keywords:** aging biology, stress, klotho, telomeres, telomerase

## Abstract

Aging biomarkers may be related to each other through direct co-regulation and/or through being regulated by common processes associated with chronological aging or stress. Klotho is an aging regulator that acts as a circulating hormone with critical involvement in regulating insulin signaling, phosphate homeostasis, oxidative stress, and age-related inflammatory functioning. Both klotho and telomere length are biomarkers of biological aging and decrease with age; however, the relationship between them is not well understood. Here we test the association between klotho levels and the telomere length of specific sorted immune cells among a healthy sample of mothers caregiving for a child with autism spectrum disorder (ASD; i.e., experiencing higher caregiving stress) or a child without ASD, covarying age and body mass index, in order to understand if high stress associated with caregiving for a child with an ASD may be involved in any association between these aging biomarkers. In 178 caregiving women (*n* = 90 high-stress mothers of children with ASD, *n* = 88 low-stress mothers of neurotypical children), we found that klotho levels were positively associated with telomere length in PBMCs (an effect driven by CD4+ and CD8+CD28− T cells) among high-stress mothers of children with an ASD but not among low-stress mothers of neurotypical children. There were no significant associations between klotho and telomerase activity in either group, across cell types assessed here. Our results suggest that klotho levels and telomere length may be associated through a coordinated downregulation of longevity factors occurring under higher stress caregiving conditions.

## INTRODUCTION

Aging biomarkers are often related to each other and this could occur through many different mechanisms. First, age influences aging related biomarkers, by definition, and any study of potential co-regulation must covary chronological age. Secondly, there may be direct co-regulation of each other. Thirdly, they may be regulated by common processes not associated with age, but associated with lifestyle factors such as chronic stress. Here we examine the relationship between two important biomarkers of aging, klotho and telomere length, in a healthy sample stratified into groups based on a combination of (a) stressor exposure and (b) level of perceived stress (i.e., high-stress mothers of children with ASD compared to low-stress mothers of neurotypical children).

Klotho is an aging regulator primarily expressed in the kidneys and choroid plexus of the brain that acts as a circulating hormone once cleaved from its transmembrane form [1–4]. Once in circulation, klotho can regulate functions of cells and tissues that do not express klotho [1, 5]. Klotho may either be overexpressed, which extends lifespan in model organisms [6], or disrupted, which accelerates systemic aging phenotypes (e.g., arterial stiffness, atherosclerosis, chronic obstructive pulmonary disease, infertility, premature mortality) [7–14]. The function of klotho is pleiotropic, with evidence supporting its role in regulating insulin signaling, phosphate homeostasis, oxidative stress, and age-related inflammatory functioning [15–20].

Like klotho, telomere length is increasingly recognized as a key biomarker of biological aging. Telomeres are protective DNA-protein complexes that cap the ends of chromosomes and provide genomic stability [21, 22]. Complete replication of telomeres is not possible due to the end replication problem of the lagging strand, which leads to telomere attrition across cell divisions. Telomeres serve as the protective ends of linear chromosomes so they are not recognized as broken ends; fortunately, telomerase, an inter-cellular enzyme, counteracts telomere attrition by adding DNA sequence repeats [23, 24]. Immune cell telomere length, typically measured in leukocytes or sorted immune cells, is consistently associated and often predictive of age-related chronic disease states, such as cardiovascular-related diseases, as well as early mortality [25–33]. In addition to age-related telomere attrition, telomeres also shorten in response to biochemical stressors (i.e., oxidative stress; [22]) and to various types of psychological stressors in a dose-response manner [34–39].

Despite evidence supporting klotho and telomere length as aging biomarkers, the relationship between them has been examined in only a handful of basic studies and is not well understood. In this regard, *in vitro* work using human cortical neurons derived from human pluripotent stem cells showed an age-related association between telomere attrition and klotho mRNA expression [40]. In another study using *in vitro* cultured cells, a 50% inhibition of klotho gene expression led to a shortening of telomere length [41], and in a separate study, the effect of impaired klotho expression on telomere attrition was found to be mediated by klotho’s regulatory effects on telomerase activity [42]. In mouse hippocampal cells, silencing klotho expression before amitriptyline treatment (an antidepressant known to induce neurotoxicity) resulted in DNA damage in telomeres due to oxidative stress and mineral imbalance, suggesting that klotho is involved in key cellular defense mechanisms that protect neurons from amitriptyline-mediated toxicity [43]. Among individuals with obstructive sleep apnea, four single genetic variants (SNPs) of the klotho gene significantly mediated the association between obstructive sleep apnea and short leukocyte telomere length [44]. When investigating relationships between klotho and telomere length among veterans, researchers identified a specific klotho SNP associated with peripheral and neuroimaging biomarkers of aging, but they did not find a significant main effect of the SNP associated with telomere length, nor did they identify a significant interaction between the SNP and PTSD severity associated with telomere length [45]. To date, no study has explored associations between sorted immune cell telomere length, telomerase activity, and circulating klotho in humans.

Chronic stress, such as the stress of caregiving for a child with a neurodevelopmental disability (e.g., autism spectrum disorder (ASD)), is associated with accelerated biological aging and the more rapid development of age-related conditions, including cardiovascular disease [46–49] and premature mortality [50, 51]. Further, chronic psychological stress has been linked to telomere attrition [52, 53], impaired telomerase activity [52, 54], and lower levels of klotho [55] raising the possibility that chronic stress may promote a coordinated downregulation in key processes implicated in longevity. To test this possibility, we explored the associations between telomere length, telomerase, and klotho in a sample of high-stress healthy women caring for a child with ASD and low-stress women caring for a neurotypical child.

## RESULTS

### Participant characteristics

Full sociodemographic characteristics of the sample (*n* = 90 high-stress mothers caring for a child with ASD, *n* = 88 low-stress mothers caring for a neurotypical child) are shown in Table 1. As previously reported [55], there were no significant differences in age, BMI, racial identity or education level between the groups. Perceived stress scores ranged from 7 to 29 in the mothers caring for a neurotypical child and 12 to 33 in the mothers caring for a child with ASD. As expected, mothers caring for a child with ASD reported significantly higher perceived stress (*M* = 21.90, *SD* = 4.69) than mothers caring for a neurotypical child (*M* = 15.73, *SD* = 4.41; *t* (174.55) = −9.03, *p* < .001). For brevity moving forward, we refer to the mothers caring for a child with ASD as the “high-stress” group and the mothers caring for a neurotypical child as the “low-stress” group.

**Table 1:** Demographic characteristics and descriptive statistics of the study participants (*N* = 178).

**Variable**	***N***	**M**	**SD**
1. Age	178	42.47	5.14
2. Education	164	16.46	2.26
3. BMI	177	25.50	5.22
4. Klotho^a^	178	891.85	324.01
5. CD4+ TL^a^	175	1.22	0.20
6. CD8+CD28+ TL^a^	175	1.24	0.24
7. CD8+CD28− TL^a^	173	1.02	0.25
8. CD19+ TL^a^	175	1.71	0.29
9. PBMC TL^a^	176	1.17	0.20
10. Granulocyte TL^a^	178	1.00	0.16
11. Whole Blood TL	173	1.08	0.16
12. PBMC TA^a^	175	5.44	3.38
13. CD4+ TA^a^	174	4.70	2.68
14. CD8+CD28+ TA^a^	169	4.63	4.84
15. CD8+CD28− TA^a^	50	2.22	1.66
16. CD19+ TA^a^	157	6.34	5.59

Abbreviations: TL: telomere length; TA: telomerase activity. ^a^Raw values presented here; values were log-transformed for analyses due to skewness.

Collapsing across groups, older age was associated with lower klotho levels (*r* = −.16, *p* = .031) and shorter telomere length across cell types (*r*s: −.20– −.37, *p*s < .01). Older age was also associated with reduced CD8+CD28+ T cell telomerase activity (*r* = −.16, *p* = .034), but there were no other significant correlations between age and telomerase activity in any other cell type (*p*s: .44–.97). Higher BMI was associated with lower klotho levels (*r* = −.15, *p* = .048) and shorter telomeres in CD19+ B cells (*r* = −.18, *p* = .02), but not shorter telomeres (*p*s: .19–.52) or telomerase activity (*p*s: .49–.95) in any other cell types. Education and race were not associated with levels of klotho, telomerase activity, or telomere length in any cell types (*p*s > .11).

### Associations of klotho levels with telomere length (TL) or telomerase activity (TA)

In models unadjusted for covariates, klotho levels were significantly associated with PBMC TL (*b* = 0.08, 95% CI (0.01, 0.15), *p* = .025) and whole blood TL (*b* = 0.21, 95% CI (0.04, 0.37), *p* = .013). When broken down into specific cell types, there were no statistically significant associations observed for CD4+ T cell TL (*b* = 0.06, 95% CI (−0.01, 0.13), *p* = .07), CD8+CD28− T cell TL (*b* = 0.10, 95% CI (−0.01, 0.21), *p* = .06), CD8+CD28+ T cell TL (*b* = 0.05, 95% CI (0.04, 0.37), *p* = .21), or CD19+ B cells (*b* = 0.04, 95% CI (−0.03, 0.12), *p* = .23). Granulocyte TL was also not significantly related to klotho levels in unadjusted models (*b* = 0.05, 95% CI (−0.02, 0.12), *p* = .15). After covarying for age and BMI, only klotho levels and whole blood TL had any signal of an association; however, this association was not statistically significant (*b* = −0.002, 95% CI (−0.02, 0.12), *p* = .052). There were no other statistically significant associations between klotho and TL across cell types in adjusted models (*p*s: .12–.67). Regarding telomerase activity (TA), there were no reliable associations between klotho levels and TA across cell types in unadjusted (*p*s: .14–.57) or adjusted (*p*s: .14–.55) models.

### Higher klotho levels associated with telomere length among high-stress, but not low-stress mothers

**Figure 1:**
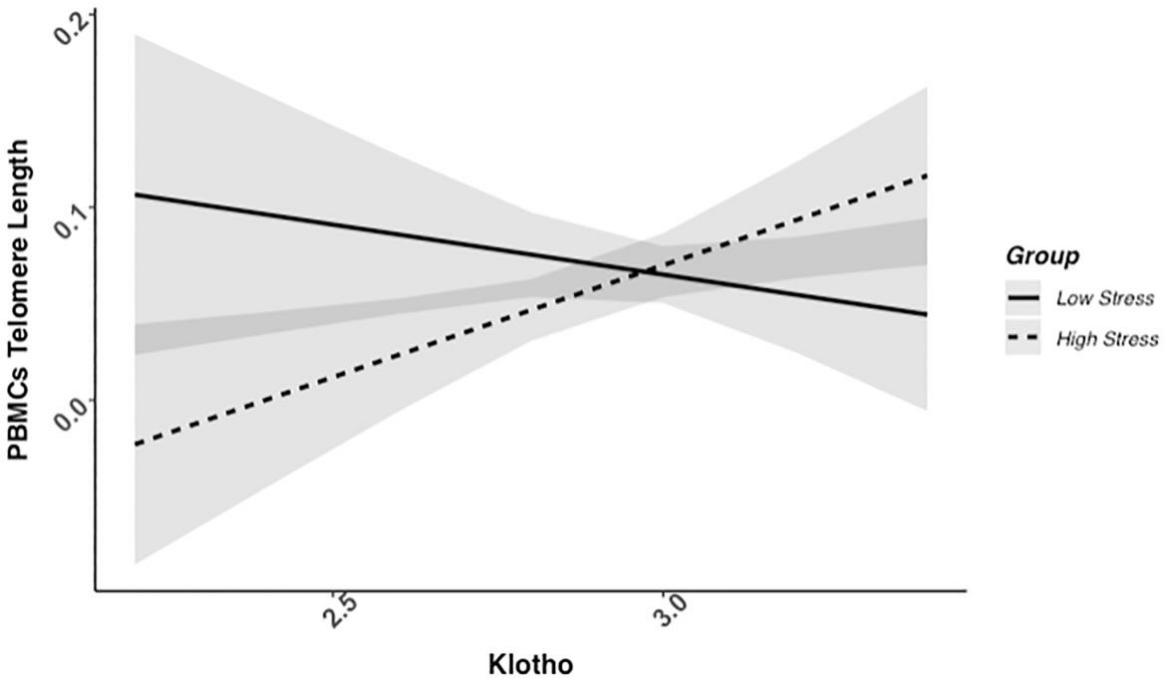
**PBMC telomere length as a function of klotho levels and stress group membership.** The slope for the high-stress group was significant, but not the slope for the low-stress group (see Table 2).

**Figure 2:**
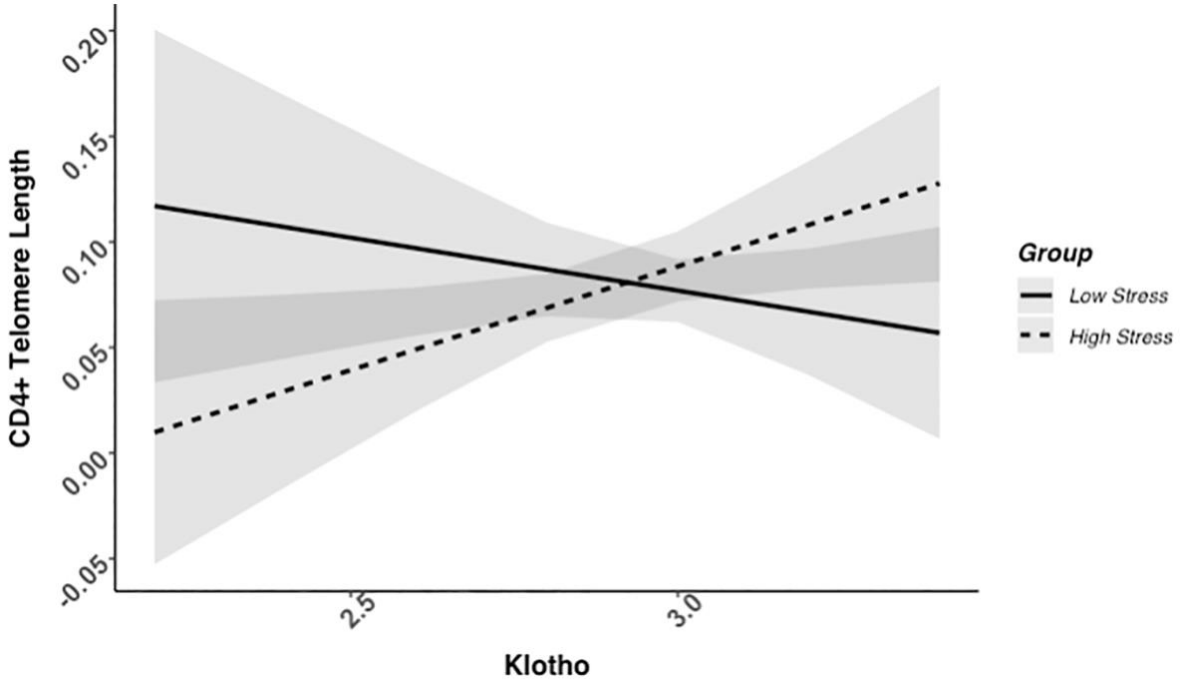
**CD4+ telomere length as a function of klotho levels and stress group membership.** The slope for the high-stress group was significant, but not the slope for the low-stress group (see Table 2).

**Figure 3:**
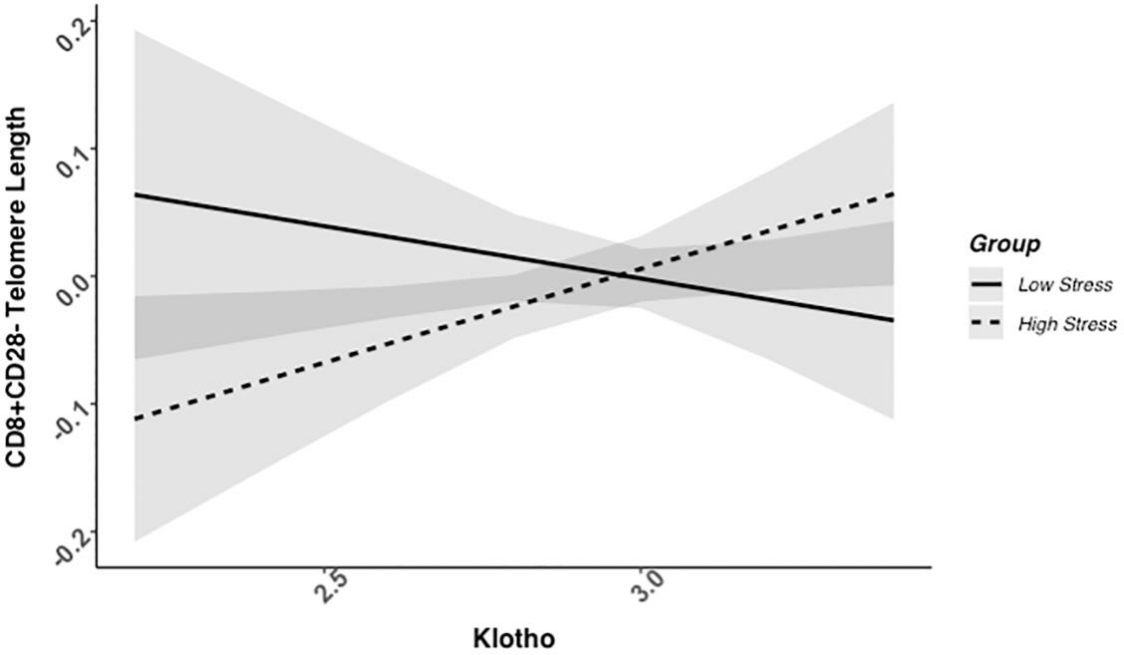
**CD8+CD28− telomere length as a function of klotho levels and stress group membership.** The slope for the high-stress group was significant, but not the slope for the low-stress group (see Table 2).

**Figure 4:**
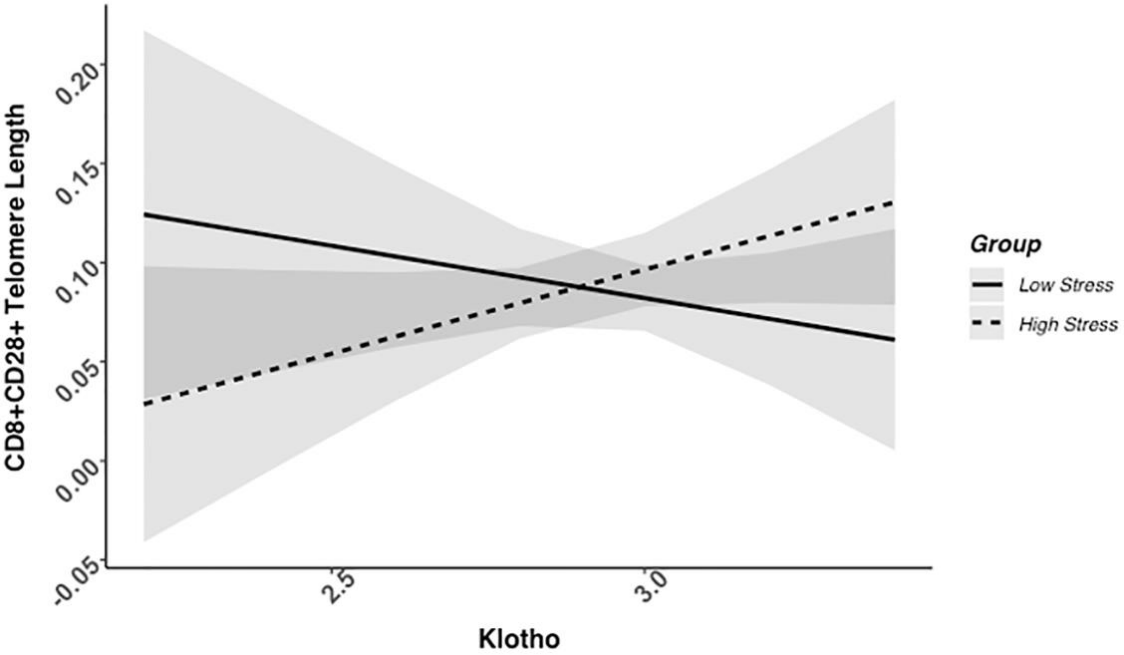
**CD8+CD28+ telomere length as a function of klotho levels and stress group membership.** Neither slope is significant in this figure (see Table 2).

We found evidence of a significant klotho by group (high- vs. low-stress) interaction for PBMC TL (unadjusted: *b* = 0.21, 95% CI (0.06, 0.35), *p* = .006; adjusted: *b* = 0.17, 95% CI (0.03, 0.31), *p* = .019; see Figure 1; see Supplementary Table 1). In an effort to understand this relationship, we explored this interaction in immune cell subsets. In this regard, we identified a significant klotho by group interaction in CD4+ T cell TL (unadjusted: *b* = 0.18, 95% CI (0.04, 0.33), *p* = .015, *p*_B-H_ = .036; adjusted: *b* = 0.15, 95% CI (0.01, 0.29), *p* = .039, *p*_B-H_ = .12; see Figure 2; see Supplementary Table 2), CD8+CD28− T cell TL (unadjusted: *b* = 0.28, 95% CI (0.06, 0.50), *p* = .014, *p*_B-H_ = .036; adjusted: *b* = 0.23, 95% CI (0.01, 0.45), *p* = .039, *p*_B-H_ = .12; see Figure 3; see Supplementary Table 3), and CD8+CD28+ T cell TL (unadjusted: *b* = 0.20, 95% CI (0.03, 0.36), *p* = .018, *p*_B-H_ = .036; adjusted: *b* = 0.14, 95% CI (−0.02, 0.29), *p* = .084, *p*_B-H_ = .17; see Figure 4; see Supplementary Table 4), but not CD19+ B cells (unadjusted: *b* = 0.08, 95% CI (−0.07, 0.23), *p* = .30, *p*_B-H_ = .30; adjusted: *b* = 0.07, 95% CI (−0.08, 0.22), *p* = .35, *p*_B-H_ = .35. There was no evidence of a significant klotho by group interaction in whole blood or for granulocyte TL (*p*s > .13).

Examination of simple slopes for the adjusted models indicated that klotho was statistically significantly associated with TL in several cell types for high-stress mothers, but not the low-stress mothers (see Table 2). Specifically, lower klotho was significantly associated with shorter TL in PBMCs, CD4+ T cells, and CD8+CD28− T cells in high- but not low-stress mothers.

**Table 2:** Simple slopes for significant interactions between klotho and stress group membership by immune cell type.

**Cell type group**	**Estimate**	**95% CI**		***p***
		**LL**	**UL**	
PBMC Telomere Length				
Low-stress	−0.04	−0.15	0.06	.41
High-stress	0.12	0.02	0.21	.019
CD4+ T Cell Telomere Length				
Low-stress	−0.05	−0.17	0.06	.36
High-stress	0.10	0.01	0.19	.029
CD8+CD28− T Cell Telomere Length				
Low-stress	−0.04	−0.21	0.13	.63
High-stress	0.15	0.01	0.29	.035
CD8+CD28+ T Cell Telomere Length				
Low-stress	−0.06	−0.17	0.05	.30
High-stress	0.08	−0.03	0.19	.15

### Associations between klotho and telomerase activity did not differ by stress group status

There was no evidence that associations between levels of klotho and cell-specific telomerase activity differed significantly between the high- and low-stress mothers. Across each cell type assessed for telomerase activity (i.e., PBMCs, CD19+ B cells, CD4+, CD8+CD28−, CD8+CD28+ T cells), there was no evidence of the klotho by group interaction associated with telomerase activity (unadjusted *p*s: .44−.74; adjusted *p*s: .44−.87).

### Sensitivity analyses

In ancillary analyses to test the robustness of these findings, we examined whether any of the main findings were driven by overly influential observations. As described next, our findings remained consistent after examining influential observations or potential outliers.

There was one significant outlier based on a Bonferroni outlier test of the studentized residuals in the analyses with CD8+CD28+ T cell TL. When this observation was removed the effect of the klotho by group interaction was similar (unadjusted: *b* = 0.20, 95% CI (0.04, 0.37), *p* = .017; adjusted: *b* = 0.14, 95% CI (−0.02, 0.30), *p* = .081). There were no overly influential observations in this regression analysis based on the DFBETAS values. There were no other overly influential observations or outliers in these analyses.

## DISCUSSION

In a sample of 178 healthy caregiving women, klotho levels were positively associated with telomere length in PBMCs for high-stress mothers caring for a child with ASD but not among low-stress mothers caring for a neurotypical child. Specifically, for high-stress mothers of a child with ASD, lower klotho levels were associated with shorter telomeres in PBMCs, with exploratory analyses highlighting that CD4+ and CD8+CD28− T cells contributed to the association across the mixed cells types. There were no significant associations between klotho and telomerase activity, nor between klotho, group (high- vs. low-stress) membership, and telomerase activity across cell types assessed here. This study is the first to explore associations between sorted immune cell telomere length, telomerase activity, and circulating klotho in humans; our results indicate that klotho and telomere length may be linked through a coordinated downregulation of certain longevity factors under higher stress caregiving conditions. Because both klotho and telomere length are associated with insulin signaling, oxidative stress, and age-related inflammatory functioning, the relationship between the klotho and telomere length may be mediated through these common stress- and longevity-related pathways.

Building on past findings in this sample that mothers of a child with ASD had lower klotho levels than mothers of a neurotypical child [55], we identified a positive relationship between klotho and telomere length among high-stress mothers of a child with ASD, but not for low-stress mothers of a neurotypical child. In a study of accelerated aging in chronic obstructive pulmonary disease (COPD) that examined interconnected hallmarks of aging [13, 56], the authors determined that both klotho levels and telomere length were decreased among COPD patients compared to controls, which supports the idea that circulating klotho is lower under disease-induced age-accelerating conditions. Mechanistically, klotho may play a role in both antagonistic and integrative hallmarks of aging by inhibiting the insulin/IGF1 signaling pathway [10, 56]. Across species, there is evidence of an evolutionarily conserved mechanism of life extension through insulin-like signaling pathways [57, 58]. Findings from model organisms that over-express klotho support klotho’s role in these signaling pathways where, in addition to inhibition of insulin/IGF1 pathways, there is also a resistance to oxidative stress that may protect against oxidative damage (Kuro-o, 2009; Kurosu et al., 2005; Yamamoto et al., 2005). Thus, under high-stress conditions, there may be broad disturbances to stress-response systems that disrupt the homeostatic balance between these interacting systems to affect aging biology as we identified here.

For high-stress mothers of a child with ASD, there was preliminary evidence that klotho may be associated with telomere length in specific sorted immune cells that are important for the aging immune system, namely T cells. As people age, telomere shortening happens primarily in CD8+ T cells [59, 60], especially among cells that have lost expression of the co-stimulatory molecule, CD28. Accordingly, CD8CD28− cells have shorter telomeres than CD8+CD28+ telomeres [61]. Critically, shorter telomeres among CD8+CD28− T cells were associated with more severe clinical symptoms among humans experimentally exposed to an upper respiratory virus [62]. A high percentage of CD8+CD28− T cells is a marker of immune senescence [63–65] and associated with poorer responses to immunization [59, 66, 67]. CD4+ T cells serve a similar function as they coordinate immune responses to pathogens [68]. Our results indicate that klotho levels and telomere length in these aged cells are linked under high-stress caregiving conditions.

We found no evidence of reliable associations between klotho and telomerase activity in this sample in either group. The impact of chronic stress itself on telomerase activity is not clear as some studies identify that chronic stress may suppress telomerase activity [52, 54, 69] while others find higher telomerase activity in combination with shorter telomeres [36, 70], which may reflect counter-regulatory attempts to combat telomere shortening occurring under chronic stress conditions. The cell type with the strongest, though still not statistically significant, evidence for an association between klotho levels and telomerase activity under high-stress was CD8+CD28− T cells, which tend to have the lowest telomerase activity of the cell types assessed here [71]. Because our sample size was smallest for this cell type (*n* = 50), this non-significant effect is worth examining in larger sample sizes.

These findings should be interpreted while recognizing the limitations of the current study design, which also highlight opportunities for important future directions. Although we focus on the stress of caregiving for a child with an ASD in this study, the caregiving experience encompasses far more than just stress [72, 73]. Because the experience of caregiving for a child with an ASD is both heterogenous and presents uniquely rewarding and joyful experiences for caregivers [72, 73], future research should not be limited to solely the stress of the caregiver but rather examine the multifaceted experience of caregiving for a child with ASD with greater focus on the strengths of each child and the structural changes that can be made to support children with ASD and reduce stress for their caregivers [72, 74]. Our specific analyses were based on cross-sectional associations and causality cannot be inferred; moreover, some analyses had smaller samples than others. For example, telomere length variables were well-represented with over 170 people for each cell type whereas there was far more variability in power to detect an effect for telomerase activity with sample sizes ranging from 50–175 across cell types. This sample included exclusively mid-life premenopausal women, so these analyses may not generalize to older or younger ages or men. Lastly, we did not control for length of caregiving or number of other children in the house, which are likely important moderators to consider in the future based on the consistent dose-response effects of chronic stress on telomere length.

These limitations are well-balanced by several key strengths of this study, including carefully matched groups of caregiving women and examining sorted immune cell telomere length. Many studies examining telomere length rely on heterogeneous populations of leukocytes without subdividing these cells; by sorting immune cells for this study we were able to identify where there were clear associations for the high-stress group between klotho and telomere length (PBMCs broadly, with some evidence for CD4+ and CD8+CD28− T cells specifically), where there was some weak evidence of an association between klotho and telomere length following the same pattern (CD8+CD28+ T cells), and where there was no evidence of an association between klotho and telomere length (CD19+ B cells, granulocytes, whole blood).

There appears to be an exciting and fruitful area for future investigations to further understand the impact of caregiving stress on aging biology. There was a wide range of power to detect an effect for telomerase activity related to group membership and klotho levels; thus, for all analyses but especially those with smaller sample sizes (e.g., CD8+ CD28− T cell telomerase activity) future work may replicate or extend these findings with larger sample sizes. Given the importance of both the chronicity and timing of stress for its impact on aging biology [75], future analyses may investigate both factors as they likely impact the associations described here. The experience of severe childhood stress, for example, is particularly impactful for stress-response system processes and may impact the magnitude of any association between stress and aging biology. Beyond characteristics of stress, future studies may also focus on the genetic component of klotho as we focused solely on soluble klotho levels.

In sum, we identified that klotho levels and telomere length of certain immune cells are linked for those experiencing higher stress caregiving conditions but not linked in lower stress caregiving conditions. This may be due to the common and powerful effects of chronic stress on stress related aging pathways. We found no evidence of this pattern of associations for klotho and telomerase activity. Future research is needed to investigate the characteristics of stress exposures that link these two markers of aging and the underlying biological mechanisms driving this connection.

## METHODS

Participants included 183 mothers recruited via schools, mailings, social media, and ads circulated through child development centers in the San Francisco Bay Area as well as direct recruitment through the University of California, San Francisco Autism Clinic; 178 of the participants had serum samples available for quantifying klotho and of those, all had DNA available for quantifying telomere length. Participants were eligible if they were non-smokers between ages 20 and 50 years, with at least one child between ages of 2 and 16 years. Participants who were caring for a child diagnosed with an autism spectrum disorder (ASD) and reported a score of *≥*13 on the Perceived Stress Scale (PSS) were categorized as a high-stress maternal caregiver. Participants who were caring for a neurotypical child (i.e., none of their children had been diagnosed with any conditions, including learning disabilities or ADHD) and reported a PSS score of *≤* 19 were categorized as a low-stress maternal control. This eligibility criteria were based on prior national norms [76, 77]. This overlapping range of PSS scores allowed participants in the low-stress group to be experiencing normal levels of stress associated with parenting that are not due to the chronic stress associated with caregiving for a child with an ASD. All study participants reported being premenopausal and in good health with no major medical conditions, such as history of coronary heart disease, endocrine disorders, epilepsy, brain injury, autoimmune conditions, severe asthma, or lung disease. Potential participants were excluded if they reported a current cancer diagnosis or had undergone chemotherapy or radiation in the past 10 years. Participants with certain current psychiatric conditions that may confound results, were excluded including individuals with bipolar disorder, post-traumatic stress disorder and eating disorders, assessed by Structured Clinical Interviews for Diagnostic and Statistical Manual for Mental Disorders for Axis I Disorders (SCID). Low-stress maternal controls with current major depression were excluded; however, this was not exclusionary for the high-stress maternal caregivers because it is a common sequela of chronic stress. No study participants were taking medications known to affect the immune and endocrine system except for antidepressant medication and oral contraceptives. Participants completed questionnaires, including sociodemographic variables, and submitted to a fasting morning blood draw at the initial study visit. This study was approved by the Institutional Review Board at the University of California, San Francisco; written, informed consent was obtained for each study participant.

### Assay of klotho

Procedures for assaying klotho have been described for this sample [55]. Briefly, serum was collected from morning fasting blood samples and stored at −80°C until assay. Soluble α-klotho was measured using a solid-phase sandwich enzyme-linked immunosorbent assay (Immuno-Biological Laboratories, Takasaki, Japan) [78], as previously described [55, 79].

### Telomere length assay

Identical methods for determining telomere length in this sample were detailed in earlier papers [80], with the exception of converting T/S ratios to base-pairs, which was not done for the analyses in this paper. Briefly, peripheral blood mononuclear cells were purified by Ficoll gradient and sorted into CD4+, CD8+CD28+, and CD8+CD28− T cells and CD19+ B cells as described previously [71]. The telomere length assay was adapted from the original method by Cawthon [71, 81]. To control for inter-assay variability, eight control DNA samples are included in each run. The T/S ratio of each control DNA was divided by the average T/S for the same DNA from 10 runs to get a normalizing factor in each batch. This was done for all eight samples and the average normalizing factor for all eight samples was used to correct the participant DNA samples to get the final T/S ratio. The T/S ratio for each sample was measured twice. When the duplicate T/S value and the initial value vary by more than 7%, the sample was run the third time and the two closest values were reported. The average coefficient of variation (CV) of this study is 2.3% (±1.8%).

### Telomerase activity assay

Telomerase activity was determined identically to how it was previously described [80]. Briefly, gel-TRAP assays were performed by the Telomerase Repeat Amplification Protocol (TRAP) using a commercial kit (TRAPeze Telomerase Detection Kit, Millipore) with modifications [71, 81]. Peripheral blood mononuclear cells were purified by Ficoll gradient and then sorted into CD4+, CD8+CD28+, and CD8+CD28− T cells and CD19+ B cells as described earlier [71]. For each telomerase activity assay reaction, the product/internal value was divided by the product/internal control value from twenty 293T cells and then multiplied by 20 to obtain the final telomerase activity units, defined as 1 unit = the amount of product from one 293T cell/10,000 immune cells. The average intra-assay variability of PBMC samples (*N* = 6, assayed in triplicate) was 8% and the inter-assay variability of PBMC samples (*N* = 24, assayed on 2 different days) was 6.7%.

### Statistical analyses

All statistical analyses were completed in the R statistical environment using R Studio (v2022.07.2). Linear regressions were performed using the *lm()* function from the *stats* package [82]. Leverage statistics for these analyses were computed using the *influence.measures()* function from the *stats* package [82]. We examined outliers using the *outlierTest()* function from the *car* package, as well as through visual examination using the *boxplot()* function [82, 83]. Plotting relied on the *ggpredict()* function from the *ggeffects* package [84], which builds on *ggplot2* capabilities [85]. Descriptive statistics were generated using the *describe()* function from the *psych* package [86]. Correlations were generated from the *cor.test()* function in the *stats* package [82].

Of the 183 participants in the parent study, 178 had serum available (missingness across relevant variables is described in Table 1). We first identified bivariate associations between sociodemographic variables and our key study variables. To examine whether the influence of klotho varied across groups, we relied on multiple linear regressions. Across primary and exploratory analyses, we first examined the interaction of interest in unadjusted models. Next, we adjusted for key covariates (age, BMI) that were associated with either klotho levels, telomere length, telomerase activity, or all three. For each exploratory model, we examined the level of significance after adjusting for multiple tests using the Benjamini-Hochberg Procedure to reduce the false-discovery rate (noted as *p*_B-H_). Lastly, sensitivity analyses tested the robustness of any significant effects by removing any potential outliers or overly influential observations. We used a Bonferonni outlier test to examine studentized residuals of each regression. Regarding leverage statistics, observations were removed if the DFBETAS value was greater than |1.0|, which indicates that a single observation may be influencing the regression parameter estimates’ values. Klotho levels underwent log-10 transformation to approximate a normal distribution. Telomere length was non-normally distributed across all cell types apart from whole blood cells; thus, we used a log-10 transformation to approximate a normal distribution for all measures of telomere length besides whole blood cells. Each measure of telomerase activity was also non-normally distributed and we applied a log-10 transformation to better approximate a normal distribution for analyses.

## Supplementary Materials

Supplementary Tables
