# Supplementary material for: Associations between klotho and telomere biology in high stress caregivers
Source: Aging (Albany NY). 2023 Aug 14;15(15):7381–96. doi: 10.18632/aging.204961 (PMC10457041; doi:10.18632/aging.204961)
Supplement: Supplementary Tables [file aging-15-204961-s001.pdf]

SUPPLEMENTARY TABLES

Supplementary Table 1. Relationship between klotho and PBMC telomere length depended on stress group membership.

	PBMC telomere length (Unadjusted)			PBMC telomere length (Adjusted)		
**Predictors**	**Estimates**	**CI**	***p***	**Estimates**	**CI**	***p***
Intercept	0.21	−0.13–0.55	0.216	0.43	0.09–0.77	**0.014**
Klotho	−0.05	−0.16–0.07	0.400	−0.05	−0.16–0.06	0.350
Group (High-stress)	−0.62	−1.05–−0.18	**0.005**	−0.50	−0.91–−0.09	**0.018**
Klotho × Group (High-stress)	0.21	0.06–0.35	**0.006**	0.17	0.03–0.31	**0.019**
Age				−0.00	−0.01–−0.00	**<0.001**
BMI				−0.00	−0.00–0.00	0.630
Observations	176			175		
R^2/R^2 adjusted	0.072/0.056			0.185/0.161		

Supplementary Table 2. Relationship between klotho and CD4+ T Cell telomere length depended on stress group membership.

	CD4+ T Cell telomere length (Unadjusted)			CD4+ T Cell telomere length (Adjusted)		
**Predictors**	**Estimates**	**CI**	***p***	**Estimates**	**CI**	***p***
Intercept	0.21	−0.12–0.55	0.215	0.42	0.08–0.77	**0.017**
Klotho	−0.04	−0.16–0.07	0.440	−0.05	−0.16–0.06	0.368
Group (High-stress)	−0.53	−0.96–−0.10	**0.015**	−0.43	−0.85–−0.02	**0.040**
Klotho × Group (High-stress)	0.18	0.04–0.33	**0.015**	0.15	0.01–0.29	**0.039**
Age				−0.00	−0.01–−0.00	**<0.001**
BMI				−0.00	−0.00–0.00	0.482
Observations	175			174		
R^2/R^2 adjusted	0.053/0.036			0.147/0.122		

Supplementary Table 3. Relationship between klotho and CD8+CD28− T Cell telomere length depended on stress group membership.

	CD8+CD28− T Cell telomere length (Unadjusted)			CD8+CD28− T Cell telomere length (Adjusted)		
**Predictors**	**Estimates**	**CI**	***p***	**Estimates**	**CI**	***p***
Intercept	0.22	−0.29–0.73	0.397	0.53	−0.00–1.06	0.051
Klotho	−0.07	−0.25–0.10	0.404	−0.08	−0.25–0.09	0.341
Group (High-stress)	−0.82	−1.47–−0.17	**0.014**	−0.68	−1.32–−0.04	**0.037**
Klotho × Group (High-stress)	0.28	0.06–0.50	**0.014**	0.23	0.01–0.45	**0.039**
Age				−0.01	−0.01–−0.00	**<0.001**
BMI				−0.00	−0.00–0.00	0.458
Observations	173			172		
R^2/R^2 adjusted	0.056/0.039			0.140/0.114		

Supplementary Table 4. Weak evidence suggesting relationship between klotho and CD8+CD28+ T Cell telomere length may depend on stress group membership.

	CD8+CD28+ T Cell Telomere Length (Unadjusted)			CD8+CD28+ T Cell Telomere Length (Adjusted)		
**Predictors**	**Estimates**	**CI**	***p***	**Estimates**	**CI**	***p***
Intercept	0.28	−0.10–0.66	0.145	0.44	0.06–0.83	**0.024**
Klotho	−0.07	−0.19–0.06	0.309	−0.05	−0.17–0.07	0.395
Group (High-stress)	−0.58	−1.06–−0.10	**0.019**	−0.40	−0.86–0.06	0.089
Klotho × Group (High-stress)	0.20	0.03–0.36	**0.018**	0.14	−0.02–0.29	0.084
Age				−0.01	−0.01–−0.00	**<0.001**
BMI				0.00	−0.00–0.00	0.249
Observations	175			174		
R^2/R^2 adjusted	0.042/0.026			0.171/0.146		
